# Systemic Inflammatory Indices in Transient Tachypnea of the Newborn: A Retrospective Case–Control Study

**DOI:** 10.3390/children12060727

**Published:** 2025-05-31

**Authors:** Mustafa Törehan Aslan, İpek Güney Varal, Gaffari Tunç, Onur Bağcı, Ayşe Ören

**Affiliations:** 1 Department of Neonatology, Koç University Hospital, 34010 Istanbul, Turkey; 2 Department of Neonatology, University of Health Sciences Bursa Yüksek İhtisas Training and Research Hospital, 16310 Bursa, Turkey; ipek.guneyvaral@sbu.edu.tr (İ.G.V.); gaffari.tunc@saglik.gov.tr (G.T.); onur.bagci1@saglik.gov.tr (O.B.); ayse.oren@sbu.edu.tr (A.Ö.)

**Keywords:** transient tachypnea of the newborn, inflammatory markers, neutrophil-to-lymphocyte ratio, systemic immune-inflammation index, gestational age

## Abstract

**Background:** Transient tachypnea of the newborn (TTN) is traditionally viewed as a disorder of delayed lung fluid clearance, but emerging evidence suggests inflammatory involvement. **Aim:** This study investigated systemic inflammatory indices [(systemic immune-inflammation index (SII-i), systemic inflammation response index (SIR-i), neutrophil-to-lymphocyte ratio (NL-r), and platelet-to-lymphocyte ratio (PL-r)] and underlying mechanisms in TTN pathogenesis for the first time. **Methods:** This retrospective case–control study included 199 neonates (123 with TTN and 76 healthy controls) admitted between 2022 and 2025 to a tertiary care hospital. Complete blood count parameters were collected within the first two hours of life. Inflammatory indices were calculated and compared between groups. Subgroup analyses were conducted based on gestational age (late preterm vs. term) and mode of delivery (cesarean vs. vaginal). **Results:** Although not statistically significant, TTN infants showed a trend toward higher inflammatory indices with median NL-r (2.54 vs. 1.75, *p* = 0.197) and SII-i (729,307.83 vs. 373,593.50, *p* = 0.276). Term TTN infants had higher NL-r (3.08 vs. 2.04, *p* = 0.022) and SII-i (729,147.74 vs. 538,928.30, *p* = 0.133) than late preterm infants. SIR-i and NL-r values were higher in the full-term group than in the early-term and late-preterm groups (*p* = 0.014, *p* = 0.022, respectively). Cesarean births showed higher NL-r (3.20 vs. 2.33, *p* = 0.049) and SII-i (*p* = 0.040) than vaginal deliveries. Strong correlations existed between SII-I, NL-r (r = 0.886, *p* < 0.01), and SII-i, SIR-i (r = 0.817, *p* < 0.01). **Conclusions:** Elevated inflammatory indices in neonates with TTN, particularly in term infants and those delivered vaginally, suggest a supportive/potential role for systemic inflammation in TTN pathophysiology. These markers may serve as potential supplementary markers for risk stratification, though further prospective validation is required to confirm their clinical relevance. These findings suggest that the early assessment of systemic inflammatory indices may assist clinicians in identifying neonates at risk for TTN, thereby guiding initial respiratory support strategies.

## 1. Introduction

Transient tachypnea of the newborn (TTN) is a prevalent respiratory system disorder affecting neonates, particularly those born via cesarean section, early term, or late preterm. It is characterized by rapid breathing (tachypnea) due to delayed clearance of fetal lung fluid, which results in respiratory distress shortly after birth [1,2]. Although TTN is typically self-limiting and resolves within 48 to 72 h, it can lead to complications such as hypoxemia and the need for respiratory support, including non-invasive mechanical ventilation, such as continuous positive airway pressure (CPAP) or supplemental oxygen. The diagnosis of TTN is primarily clinical, relying on symptoms such as grunting, nasal flaring, and retractions, but differentiating it from other respiratory conditions like respiratory distress syndrome (RDS) or pneumonia remains a challenge [1,2,3]. Understanding the underlying mechanisms and identifying reliable biomarkers for TTN could improve early diagnosis and management [1,4,5]. TTN severity is commonly classified by respiratory support needs: mild (room air), moderate (only oxygen therapy), or severe (non-invasive ventilation support) [1,4,5,6].

In recent years, systemic inflammatory index markers such as the NL-r, PL-r, and SII-i have gained attention as potential indicators of inflammation and immune response in various medical conditions [7,8,9]. These markers are derived from routine complete blood count (CBC) tests, making them cost-effective and easily accessible. NL-r reflects the balance between immune systems (innate–adaptive), while PL-r and SII-i provide insights into platelet activity and systemic inflammation [8,9]. In neonates, these markers have been explored in conditions such as sepsis, neonatal pneumonia, and intrauterine growth restriction, but their role in TTN remains underexplored [10,11]. Given the inflammatory component associated with delayed lung fluid clearance in TTN, these markers could offer valuable insights into the pathophysiology of the condition.

The association between TTN and systemic inflammatory index markers such as NL-r, PL-r, and SII-i is an emerging area of research. Inflammation plays a critical role in the pathogenesis of TTN, as the delayed absorption of fetal lung fluid may initiate an immune response, leading to the release of proinflammatory cytokines and the activation of neutrophils [1,2,4]. Elevated NL-r, PL-r, and SII-i levels have been linked to increased inflammation in various neonatal conditions, suggesting their potential utility in assessing the severity and prognosis of TTN. Investigating these markers in TTN could not only enhance our understanding of the disease but also provide a non-invasive tool for early diagnosis and monitoring. This study specifically targeted neonates with severe transient tachypnea of the newborn (TTN), operationally defined by the requirement for nasal continuous positive airway pressure (nCPAP) due to clinically significant respiratory distress. As such, the findings are most applicable to the subset of TTN cases presenting with a more pronounced clinical course, rather than encompassing the entire spectrum of TTN severity. By restricting the cohort to infants necessitating non-invasive respiratory support, the study aimed to more accurately characterize the inflammatory profile associated with moderate-to-severe disease manifestations.

## 2. Methods

### 2.1. Study Plan

This study was designed as a retrospective case–control study conducted between 2022 and 2025. The primary objective was to investigate the association between TTN and systemic inflammatory index markers, including NL-r, PL-r, SII-i, and SIR-i. This study aimed to compare these markers between neonates diagnosed with TTN and healthy controls to evaluate their diagnostic and prognostic potential.

### 2.2. Study Population

The study population consisted of neonates in the neonatal department of Bursa Yüksek İhtisas Training and Research Hospital in Bursa, Turkey during the study period. A total of 199 neonates were included in the study. A total of 123 neonates born between 34 + 0/7 and 40 + 6/7 weeks of gestation admitted to the neonatal intensive care unit due to TTN and 76 healthy neonates (control group) matched for gestational age, delivery type (vaginal delivery or cesarean section), and birth weight were included in the study. Cases were defined as neonates diagnosed with TTN based on clinical criteria, including tachypnea (a respiratory rate higher than 60), retractions, grunting, and nasal flaring along with radiographic findings consistent with TTN. To standardize disease severity and eliminate confounding factors in mild cases managed without respiratory support, inclusion was restricted to TTN infants requiring nasal CPAP support (severe TTN) [6].

In our study, patients with TTN were selected as those requiring only non-invasive ventilation support (nasal continuous positive airway pressure); patients who underwent mode changes, were intubated, or had any other problems during treatment were not included in the study.

Controls were healthy neonates matched for gestational age, birth weight, and mode of delivery (vaginal or cesarean section). Healthy neonates whose mothers were healthy and who underwent blood tests for blood type incompatibility in the well-baby ward next to their mothers were included in the study as a control group. None of the neonates in the control group required NICU admission.

Exclusion criteria included neonates with congenital anomalies, neonatal sepsis, pneumonia, respiratory distress syndrome (RDS), or other significant comorbidities that could influence inflammatory markers. Sepsis was excluded through combined clinical assessment (absence of sepsis symptoms), laboratory markers (CBC, CRP, and cultures), and clinical course (rapid TTN resolution without antibiotic needs beyond 48 h). Those born at or after 41 + 0/7 weeks of gestation and those born before 34 + 0/7 weeks of gestation were not included in the study.

### 2.3. Data Collection

Data were collected retrospectively from the hospital medical records database, including demographic information (gestational age, birth weight, sex, and mode of delivery), clinical parameters (respiratory rate, oxygen requirement, and duration of respiratory support), and laboratory findings (complete blood count with differential). Patients were divided into 3 groups according to gestational age: late preterm (between 34 + 0/7 and 36 + 6/7 weeks of gestation), early term (between 37 + 0/7 and 38 + 6/7 weeks of gestation), and full term (between 39 + 0/7 and 40 + 6/7 weeks of gestation). To avoid the potential confounding effects of respiratory support on inflammatory markers, blood samples were collected before the initiation of nasal continuous positive airway pressure (nCPAP), within the first two hours of life. In most cases, sampling occurred upon admission to the neonatal intensive care unit (NICU), with a median collection time of 30 min after birth. Importantly, all samples were obtained prior to the start of any respiratory intervention. Complete blood counts, including differential parameters, were performed within one hour of collection. Samples were drawn into EDTA-anticoagulated tubes and analyzed using an automated hematology analyzer (Mindray BC-6000, Mindray Bio-Medical Electronics Co., Ltd., Shenzhen, China). The device was calibrated daily using quality control materials provided by the manufacturer, and all analyses were conducted according to standardized laboratory protocols to ensure accuracy and reliability. Gestational age was primarily determined via first-trimester ultrasonography performed before 14 weeks of gestation. In cases where ultrasound data were not available, maternal recall of the last menstrual period was used to estimate gestational age.

### 2.4. Systemic Inflammatory Index Markers (SII-i, SIR-i, NL-r, and PL-r)

Systemic inflammatory index markers were derived from routine CBC tests performed within the first two hours of life. These markers were chosen for their ability to reflect systemic inflammation and immune response. NL-r and PL-r are well-established indicators of inflammation, while SII-i and SIR-i provide a more comprehensive assessment of immune activation by incorporating platelet and monocyte counts, respectively.

Systemic inflammatory index markers were calculated using the following formulas [8,12]:

- NL-r = neutrophil–lymphocyte counts;
- PL-r = platelet–lymphocyte counts;
- SII-i = (neutrophil×platelet)–lymphocyte counts;
- SIR-i = (neutrophil×monocyte)–lymphocyte counts.

### 2.5. Ethics Approval

The study protocol was approved by the Medical Sciences Ethics Committee of the University of Health Sciences, Bursa Yüksek İhtisas Training and Research Hospital with the date and number 14 August 2024 and 2024-TBEK 2024/08-08. All procedures were carried out in accordance with the ethical standards of the Declaration of Helsinki.

### 2.6. Statistical Analyses

Statistical analyses were performed using the SPSS software (version 25.0). Continuous variables were expressed as median (25–75 interquartile range); categorical variables are presented as frequencies and percentages. Comparisons between TTN cases and controls were made using the Mann–Whitney U test for continuous variables and the chi-square test for categorical variables. Non-parametric tests (Mann–Whitney U and Kruskal–Wallis) were used for non-normally distributed data, and multiple comparisons were adjusted via Bonferroni correction where applicable. Correlations between inflammatory markers and clinical outcomes were assessed using Spearman correlation coefficients. A *p*-value < 0.05 was considered statistically significant. Independent variables included in the logistic regression model were delivery mode, gestational age, birth weight, sex, Apgar scores (1st and 5th min), and inflammatory indices (NL-r, PL-r, SII-i, and SIR-i). We used binary logistic regression analysis to evaluate the factors (gender, birth weight, gestational age, Apgar scores, NL-r, PL-r, SII-i, and SIR-i) affecting TTN. The logistic regression model included gender, birth weight, gestational age, Apgar scores at 1st and 5th min, mode of delivery, and inflammatory indices (NL-r, PL-r, SII-i, and SIR-i) as independent variables. These variables were selected based on clinical relevance and their potential association with TTN risk.

## 3. Results

### 3.1. Demographic and Clinical Characteristics

Of the patients included in this study, 61.8% (*n* = 123) were in the TTN group, while 38.2% (*n* = 76) were in the control group. Most of the patients were delivered via cesarean section (C/S), with 74.4% (*n* = 148) of the total cohort (*n* = 199) born via C/S, while 25.6% (*n* = 51) were delivered vaginally. Of the cesarean deliveries, constituting 74% of the cohort, 82% were elective (without labor), and 18% were emergency procedures (after labor onset), as documented in maternal records. In terms of gender distribution, 60.3% (*n* = 120) of the participants were male, and 39.7% (*n* = 79) were female. The median gestational age was 37 weeks, and the median birth weight was 2912.31 g (Table 1).

### 3.2. Comparison of Inflammatory Markers Between TTN and Control Groups

The systemic inflammatory index markers, including NL-r, PL-r, SII-i, and SIR-i, were compared between the TTN and control groups. The median NL-r in the TTN group was 2.54 (1.54–3.81), compared to 1.75 (1.13–3.10) in the control group (*p* = 0.197). Similarly, the median PL-r was 74.18 in the TTN group and 59.55 in the control group (*p* = 0.558). The SII-i and SIR-i values were also higher in the TTN group, with median values of 729,307.83 and 3048.50, respectively, compared to 373,593.50 and 1590.65 in the control group (*p* = 0.276 and 0.332, respectively) (Table 2).

### 3.3. Subgroup Analysis Based on Delivery Mode

In the subgroup analysis, patients delivered via C/S had significantly lower NL-r and SII-i values compared to those delivered vaginally (*p* = 0.049 and 0.040, respectively). The median NL-r in the C/S group was 2.32 (1.38–3.61), compared to 3.20 (1.70–4.49) in the vaginal delivery group. Similarly, the median SII-i was 645 in the C/S group and 936 in the vaginal delivery group (*p* < 0.05). No significant difference was found between the groups in other hematological parameters (Table 2).

### 3.4. Comparison of Late Preterm and Term Infants in the TTN Group

In the TTN group, a subgroup analysis was conducted to compare systemic inflammatory index markers between late preterm (gestational age between 34 + 0/7 and 36 + 6/7 weeks) and term infants (early term and full term) (gestational age between 37 + 0/7 and 40 + 6/7 weeks). The median SII-i in late preterm infants was 539, lower than the median SII-i of 792 in term infants (*p* = 0.110). The median SIR-i in late preterm infants was 2.2 (1.2–4.2), compared to 3.9 (1.6–6.7) in term infants (*p* = 0.046). Similarly, a higher median value, 6.1 (3.5–10.3), was found in full-term infants, higher than in the early-term and late-preterm infant groups (*p* = 0.014). For NL-r, late-preterm infants had a median value of 2.04 (1.17–3.28), while term infants exhibited a higher median NL-r of 3.08 (1.88–4.0) (*p* = 0.016). Similarly, the median NL-r value, 3.9 (2.2–5.2), in full-term infants was higher than in the early-term and late-preterm infant groups (*p* = 0.022), and the median PL-r in late-preterm infants was 67.60 (54.70–94.0), which was lower than the median PL-r of 76.29 (61.19–92.18) in term infants, though this difference was not statistically significant (*p* = 0.403) (Table 3).

### 3.5. Correlation Between Clinical Outcomes and Inflammatory Markers

Spearman’s correlation analysis revealed significant associations between inflammatory markers and clinical parameters. A strong positive correlation was observed between SII-i and NL-r (r = 0.810, *p* < 0.01) and between SII-i and SIR-i (r = 0.745, *p* < 0.01). Additionally, Apgar scores (first minute) showed a moderate positive correlation with NL-r (r = 0.269, *p* = 0.004), while Apgar scores (fifth minute) were positively correlated with NL-r (r = 0.311, *p* = 0.001). However, no significant correlation was found between inflammatory markers and birth weight or gestational age (Table 4).

### 3.6. Regression Analyses

In the logistic regression analysis (Table 5), after adjusting for potential confounders, including gender, birth weight, gestational age, Apgar scores (1st and 5th mins), NL-r, PL-r, SII, and SIRI, cesarean section emerged as an independent predictor of TTN (AOR = 3.78; 95% CI: 2.40–80.8; *p* = 0.011). However, the considerable breadth of the confidence interval indicates limited precision in the risk estimate, potentially attributable to sample size limitations or distributional imbalance between comparison groups. Thus, while the association reached statistical significance, the result warrants cautious interpretation and should ideally be confirmed in larger, prospectively designed studies.

## 4. Discussion

To the best of our knowledge, this is the first study in the literature to evaluate systemic inflammatory markers SII-i, SIR-i, NL-r, and PL-r in neonates with TTN. Term infants exhibited a higher median NL-r of 3.08 (1.88–4.0) (*p* = 0.022). The patients delivered via C/S had significantly lower NL-r and SII-i values compared to those delivered vaginally (*p* = 0.049 and 0.040, respectively). Apgar scores (first minute) showed a moderate positive correlation with NL-r (r = 0.269, *p* = 0.004), while Apgar scores (fifth minute) were positively correlated with NL-r (r = 0.311, *p* = 0.001). The most striking results of our study were that we found that SIR-i and NL-r values were higher in full-term infants than in the early-term and late-preterm groups (*p* = 0.014, *p* = 0.022, respectively).

Although the differences did not reach statistical significance (SII-i: *p* = 0.276; SIR-i: *p* = 0.332), both indices were higher in neonates with TTN compared to controls, which was suggestive but not conclusive. This trend is consistent with previous studies proposing that oxidative stress resulting from delayed lung fluid clearance may contribute to the inflammatory response observed in TTN [1,2]. Several studies investigating neonatal respiratory distress syndrome (RDS) and pneumonia have reported elevated SII-i levels in affected infants compared to controls, supporting the potential role of systemic inflammation in the pathogenesis of these conditions [10,13]. SII-i, which integrates neutrophils, platelets, and lymphocytes, may reflect a broader inflammatory state, while SIR-i’s inclusion of monocytes could indicate macrophage involvement in lung injury [13,14]. Further research with larger cohorts is needed to confirm these findings and explore whether SII-i/SIR-i could serve as prognostic markers for TTN severity.

No statistically significant differences in NL-r or PL-r were observed between neonates with TTN and healthy controls. To the best of our knowledge, no previous studies have specifically examined NL-r or PL-r in the context of TTN. However, a study investigating respiratory distress syndrome (RDS) reported elevated NL-r levels in affected neonates, suggesting a potential link between inflammatory markers and more severe forms of neonatal respiratory distress [13]. In a study conducted by Wang et al. [10] on neonatal pneumonia, it was stated that NL-r and PL-r values increased, similar to the RDS study. The lack of significance in PL-r (*p* = 0.558) contrasts with some studies on neonatal respiratory conditions like neonatal pneumonia and RDS where elevated PL-r was observed [10,13]. Hypothetically, NL-R’s stronger association may stem from its reflection of acute-phase inflammation, whereas PL-R’s variability could relate to platelet dynamics in neonatal sepsis or other confounders. Future studies should standardize the timing of blood sample collection to minimize the effects of circadian rhythms—natural physiological fluctuations that can influence leukocyte and platelet levels in neonates due to endogenous hormonal cycles, thereby potentially affecting calculated inflammatory indices that occur over a 24 h cycle—which can influence immune cell counts and potentially affect the accuracy of inflammatory indices like NL-r and PL-r.

Full-term infants with TTN exhibited higher NL-r (*p* = 0.022) and trends toward elevated SIR-i (*p* = 0.014) compared to late-preterm infants, suggesting gestational age modulates inflammatory responses. A previous study reported that the neutrophil-to-lymphocyte ratio (NL-r) and systemic immune-inflammation index (SII-i) were significantly elevated in the preterm birth group, suggesting the potential involvement of systemic inflammation in the development of preterm labor [15]. Term neonates may mount a more robust immune reaction due to mature cytokine pathways, whereas the late preterms’ immature immunity could attenuate inflammation [16,17,18]. SIRI was significantly higher in full-term infants, indicating a gestational age-related modulation of inflammatory response. While systemic inflammatory indices were highest in full-term infants, this pattern was primarily observed in elective cesarean deliveries without labor (82% of C/S cases). This suggests that the inflammatory response may reflect interrupted physiological preparation for birth, though vaginal delivery cases require separate consideration due to differing hormonal dynamics [19,20].

Cesarean-delivered neonates demonstrated significantly lower NL-r and SII-i values compared to those born vaginally (*p* = 0.049 and *p* = 0.040, respectively). Of the cesarean deliveries, constituting 74% of the cohort, 82% were elective procedures performed before the onset of labor. This high rate of elective cesarean sections, by bypassing labor-induced hormonal and inflammatory signaling, may impair fetal lung fluid clearance and thereby contribute to the increased incidence of TTN observed in this cohort—highlighting a possible mechanistic link that aligns with, rather than contradicts, existing hypotheses. Although logistic regression analysis indicated that cesarean section significantly increased the risk of TTN, the wide confidence interval (2.40–80.8) highlights considerable uncertainty in the strength of this association. This may reflect limitations related to sample size or group imbalance and suggests the need for further validation in larger studies. A plausible explanation may lie in the heightened inflammatory response associated with vaginal birth, potentially triggered by mechanical stress during passage through the birth canal or early microbial exposure. On the other hand, the attenuation of inflammatory markers in cesarean-born infants may reflect confounding influences such as elective delivery prior to the onset of labor or maternal antibiotic administration—both known to modulate neonatal immune activity [21,22]. These observations emphasize the complex interplay between the mode of delivery and neonatal immune regulation. Further prospective studies, carefully controlling for labor duration, maternal intrapartum factors, and antibiotic use, are warranted to delineate the mechanisms underlying these associations. Our findings also support the concept that elective cesarean section, when performed in the absence of labor, may alter the early inflammatory environment of the newborn by limiting exposure to immunological stimuli typically encountered during vaginal birth, including hormonal surges and vertical microbiota transmission [23,24]. In our cohort, male neonates accounted for 60.3% of the cases, consistent with previous studies identifying male sex as a risk factor for TTN [25,26]. However, despite this predominance, no statistically significant differences in inflammatory indices (NL-r, PL-r, and SII-i) were observed between sexes. This suggests that systemic inflammation alone may not fully explain the increased susceptibility observed in male neonates. Nonetheless, the slightly higher median NL-r in males (2.60 vs. 2.45) could reflect subtle sex-based immunological differences, potentially influenced by prenatal androgen exposure. Although not statistically significant in our study, this trend merits further investigation in larger, multicenter cohorts.

Strengths and Limitations

This study is the first in the literature to systematically evaluate systemic inflammatory markers in a well-defined and homogenous cohort of neonates diagnosed with transient tachypnea of the newborn (TTN). This is a significant contribution to the field. Including only severe TTN cases requiring nasal CPAP support strengthens the internal validity of our findings and highlights the potential link between inflammation and clinically significant TTN. However, this also limits the external validity, as the results may not be generalizable to milder forms of TTN. Because we included only TTN cases requiring non-invasive ventilation, the findings predominantly reflect the inflammatory status of severe TTN presentations. This selection criterion limits the applicability of our results to milder cases that may follow a different inflammatory profile. Due to the retrospective design, no post-discharge follow-up data were available. Future prospective studies with longitudinal outcomes are needed to understand the long-term impact of elevated inflammatory markers. The relatively limited sample size represents a constraint, potentially affecting the statistical power of the subgroup analyses. Furthermore, while additional inflammatory markers such as IL-6, procalcitonin, and neutrophil maturation indices would have enriched the dataset, these are not routinely incorporated into neonatal sepsis evaluations in our clinical setting. IL-6 is not commonly available (it is neither cost-effective nor cost-saving), and procalcitonin is known to exhibit physiological elevations in the early postnatal period (it may sometimes lead to confusion regarding the prediction of early-onset neonatal sepsis), limiting its diagnostic utility. These factors reflect real-world clinical practice and institutional protocols; however, they also highlight the need for multicenter, prospective studies with broader biomarker panels and larger populations to confirm and expand the findings of our study, drawing attention to this unique topic for the first time.

## 5. Conclusions

This study provides the first comprehensive evaluation of systemic inflammatory indices—SII-i, SIR-i, NL-r, and PL-r—in neonates with TTN. Our findings demonstrate that cesarean delivery is significantly associated with an increased risk of TTN. Interestingly, inflammatory markers such as NL-r and SII-i were found to be lower in cesarean-delivered neonates compared to those born vaginally, suggesting that the inflammatory profile may be influenced by the mode of delivery, potentially due to the absence of labor-related physiological stimuli. In addition, higher NL-r and SIR-i values observed in full-term infants compared to late-preterm and early-term groups point toward the possible role of gestational maturation in modulating systemic inflammatory responses. These results underscore the multifactorial nature of TTN and highlight the need for further prospective studies to validate the clinical utility of these markers in risk stratification and early diagnosis.

## Figures and Tables

**Table 1:** Evaluation of demographic and hematological characteristics.

**Features**	**% (n)**
Group	
TTN *	61.8 (123)
Control	38.2 (76)
Gender	
Male	60.3 (120)
Female	39.7 (79)
Delivery Mode	
C/S **	74.4 (148)
Vaginal delivery	25.6 (51)
**Variable**	**Median (25–75 IQR)**
Gestational age (weeks)	37 (36–38)
Apgar score (1st min)	8 (8–9)
Apgar score (5th min)	9 (9–10)
Birth weight (grams)	2890 (2565–3205)
NIV *** requirement (hour)	24 (10–48)
Maximum FiO_2_ ****	25 (21–30)
Leukocytes (×10^3^/μL)	14.9 (11.7–19.3)
Neutrophils (×10^3^/μL)	8.9 (5.4–12.8)
Lymphocytes (×10^3^/μL)	4.1 (3.2–4.8)
Platelets (×10^3^/μL)	304 (250–353)
NLR	2.50 (1.43–3.80)
PLR	74.18 (59.55–93.33)
SII ^ɸ^ (×10^3^)	709 (422–1070)
SIRI ^§^ (×10^3^)	2.9 (1.3–5.9)

* = Transient tachypnea of the newborn, ** = cesarean section, *** = non-invasive ventilation, **** = fraction of inspired oxygen, ^ɸ^ = systemic inflammatory index, and ^§^ = systemic inflammation response index.

**Table 2:** Comparison of demographic and hematological characteristics in transient tachypnea of the newborn (TTN)–control and vaginal delivery–C/S groups.

Variable	TTN Group n = 123 ^з^	Control Group n = 76 ^з^	p1 *	Vaginal Delivery n = 51 ^з^	C/S n = 148 ^з^	p2 ^¥^
Gestational age (weeks)	37 (36–38)	38 (36–39)	0.280	37 (36–38)	38 (36–40)	**0.003**
Birth weight (grams)	2860 (2550–3190)	2972 (2705–3372)	0.130	2845 (2553–3190)	2985 (2640–3330)	0.119
Apgar score 1st min	8 (7–9)	8 (7–9)	0.564	8 (7–9)	8 (8–9)	**0.004**
Apgar score 5th min	9 (9–10)	9 (9–10)	0.936	9 (9–10)	10 (9–10)	**0.034**
Leukocytes (×10^3^/μL)	16.4 (12.9–20.4)	12.5 (10.2–16.0)	**<0.001**	15.1 (11.9–18.9)	14.9 (10.5–22.0)	0.889
Neutrophils (×10^3^/μL)	10.1 (6.7–13.6)	7.4 (4.1–11.4)	**0.001**	8.8 (5.5–12.5)	10.5 (4.7–15.8)	0.417
Lymphocytes (×10^3^/μL)	4.1 (3.1–4.9)	4.0 (3.3–4.7)	0.916	4.2 (3.3–4.9)	4.0 (3.7–4.5)	0.090
Platelets (×10^3^/μL)	305 (257–348)	297 (244–360)	0.609	306 (256–357)	287 (244–346)	0.198
NLR	2.54 (1.54–3.81)	1.75 (1.13–3.10)	0.197	2.32 (1.38–3.61)	3.20 (1.70–4.49)	**0.049**
PLR	72.2 (59.7–93.3)	87.75 (54.9–97.7)	0.558	71.6 (59.1–87.6)	83.54 (60.4–97.9)	0.224
SII ^ɸ^ (×10^3^)	729 (428–1071)	374 (230–1286)	0.276	645 (396–1059)	936 (438–1558)	**0.040**
SIRI ^§^ (×10^3^)	3.1 (1.4–6.0)	1.6 (0.6–7.4)	0.332	2.6 (1.3–4.5)	4.6 (2.1–7.3)	**0.049**

* Mann–Whitney U test was performed between TTN and control groups; ^з^ data: median (25–75 IQR); *p* < 0.05 significant. ^ɸ^ = systemic inflammatory index; ^§^ = systemic inflammation response index; ^¥^ = Mann–Whitney U test was performed between C/S (cesarean section) and vaginal delivery groups.

**Table 3:** Evaluation of demographic, hematological characteristics, and systemic inflammatory markers in the transient tachypnea of the newborn (TTN) group according to gestational age grouping.

Variable	Late Preterm n = 40 ^з^	Early Term n = 61 ^з^	Full Term n = 22 ^з^	All Term n = 83 ^з^	p1 *	p2 **
Gestational age (weeks)	35 (35–36)	37 (37–38)	39 (39–40)	38 (37–40)	**<0.001**	**<0.001**
Birth weight (grams)	2582 (2357–2860)	2900 (2700–3180)	3313 (2920–3725)	2945 (2760–3295)	**<0.001**	**<0.001**
Apgar score (1st min)	8 (7–8)	8 (8–9)	8.5 (7.8–9)	8 (8–9)	**0.006**	**0.024**
Apgar score (5th min)	9 (9–10)	9 (9–10)	9.5 (9–10)	10 (9–10)	**0.016**	0.059
NIV *** requirement (hour)	24 (13–72)	24 (9.5–48)	24 (12–48)	24 (10–48)	0.511	0.768
Maximum FiO_2_ ****	25 (21–30)	25 (21–30)	25 (21–30)	25 (21–30)	0.606	0.876
Leukocytes (×10^3^/μL)	15.4 (12.1–18.3)	17.0 (13.0–20.1)	20.2 (14.0–24.3)	17.5 (13.4–20.9)	**0.044**	**0.013**
Neutrophils (×10^3^/μL)	8.9 (6.1–11.7)	10.7 (6.9–13.9)	13.3 (8.3–16.8)	11.3 (7.0–15.0)	**0.037**	**0.033**
Lymphocytes (×10^3^/μL)	4.1 (3.1–5.1)	4.0 (3.1–4.7)	4.3 (3.4–4.9)	4.1 (3.1–4.9)	0.580	0.717
Platelets (×10^3^/μL)	311 (244–384)	289 (258–334)	315 (255–359)	290 (257–341)	0.355	0.387
NLR	2.04 (1.17–3.28)	2.58 (1.76–3.80)	3.9 (2.2–5.2)	3.08 (1.88–4.0)	**0.016**	**0.022**
PLR	67.6 (54.7–94.0)	79.6 (63.2–93.3)	65.2 (58.6–91.1)	76.3 (61.2–92.2)	0.358	0.451
SII ^ɸ^ (×10^3^)	539 (316–1072)	779 (528–1069)	894 (426–1157)	792 (520–1070)	0.110	0.312
SIRI ^§^ (×10^3^)	2.2 (1.2–4.2)	2.7 (1.6–6.0)	6.1 (3.5–10.3)	3.9 (1.6–6.7)	**0.046**	**0.014**

* Mann–Whitney U test was performed between late-preterm and term groups. Data: Median (25–75 IQR); *p* < 0.05 significant. ** = Kruskal–Wallis test was performed between late-preterm, early-term, and full-term groups. ^з^ Data: Median (25–75 IQR); *p* < 0.05 significant; ^ɸ^ = systemic inflammatory index; ^§^ = systemic inflammation response index; *** = non-invasive ventilation; **** = fraction of inspired oxygen.

**Table 4:** Correlation of birth weight, Apgar scores, NIV requirement, maximum FiO_2_, and systemic inflammatory index markers.

		Birth Weight	Apgar Score (1st Min)	Apgar Score (5th Min)	NLR	PLR	SII	SIRI	NIV Requirement ***	Maximum FiO_2_ ****
Birth weight	Cor. Coef. ^¥^ Sig N	1 123								
Apgar score (1st min)	Cor. Coef. Sig N	**0.225** 0.012 * **123**	1 123							
Apgar score (5th min)	Cor. Coef. Sig N	0.168 0.063 123	0.882 ** <0.001 123	1 123						
NL-r	Cor. Coef. Sig N	0.188 0.058 123	0.269 ** 0.004 123	**0.311** **0.001** 123	1 123					
PL-r	Cor. Coef. Sig N	−0.056 0.573 123	−0.103 0.299 123	0.057 0.569 123	0.277 ** **0.005** **123**	1 123				
SI-i	Cor. Coef. Sig N	0.092 0.355 123	0.107 0.284 123	0.197 * **0.046** **123**	0.810 ** <0.001 123	0.484 ** <0.001 123	1 123			
SIR-i	Cor. Coef. Sig N	0.180 0.068 123	0.185 0.062 123	0.176 * 0.076 123	0.891 ** <0.001 123	0.138 0.164 123	0.745 ** <0.001 123	1 123		
NIV requirement ***	Cor. Coef. Sig N	−0.147 0.104 123	−0.179 * **0.048** **123**	−0.116 0.201 123	**0.017** **0.867** 123	0.150 0.132 123	**0.059** **0.554** 123	0.009 0.927 123	1 123	
Maximum FiO_2_	Cor. Coef. Sig N	−0.082 0.370 123	−0.023 0.800 123	−0.053 0.559 123	−0.154 0.121 123	0.056 0.577 123	−0.174 0.078 123	−0.166 0.094 123	0.212 * **0.018** **123**	1 123

* Correlation is significant at the <0.05 level; ** correlation is significant at the 0.01 level (2-tailed); *** = non-invasive ventilation; **** = fraction of inspired oxygen; ^¥^ = Pearson correlation coefficient.

**Table 5:** Logistic regression model evaluating the association between demographic, perinatal, and inflammatory variables and the risk of TTN.

	AOR	95% CI	*p*
Delivery Mode	3.78	2.40–8.08	**0.011**
Birth weight	1.00	0.99–1.01	0.616
Apgar score (1st min)	1.52	0.89–2.28	0.517
Apgar score (5th min)	0.84	0.56–1.26	0.944
NL-r	1.36	0.37–1.76	0.166
PL-r	1.00	0.96–1.04	0.930
SII-i	0.98	0.95–1.01	0.811
SIR-i	1.00	0.99–1.01	0.212
Gender	0.02	0.01–0.10	0.956

AOR = adjusted odds ratio; CI = confidence interval; NL-r = neutrophil-to-lymphocyte ratio; PL-r = platelet-to-lymphocyte ratio; SII-i = systemic immune-inflammation index; SIR-i = systemic inflammation response index. *p*-values are adjusted for all variables listed. Bold values indicate statistical significance (*p* < 0.05).

## Data Availability

The research data are not publicly available.
